# Frequency detection, frequency discrimination, and spectro-temporal pattern perception in older and younger typically hearing adults

**DOI:** 10.1016/j.heliyon.2023.e18922

**Published:** 2023-08-06

**Authors:** Duo-Duo Tao, Bin Shi, John J. Galvin, Ji-Sheng Liu, Qian-Jie Fu

**Affiliations:** a Department of Ear, Nose, and Throat, The First Affiliated Hospital of Soochow University, Suzhou, 215006, China; b House Institute Foundation, Los Angeles, CA, 90057, USA; c University Hospital Center of Tours, Tours, 37000, France; d Department of Head and Neck Surgery, David Geffen School of Medicine, University of California, Los Angeles, CA, 90095, USA

**Keywords:** Aging, Spectro-temporal modulated ripple test, Frequency discrimination, Elderly

## Abstract

Elderly adults often experience difficulties in speech understanding, possibly due to age-related deficits in frequency perception. It is unclear whether age-related deficits in frequency perception differ between the apical or basal regions of the cochlea. It is also unclear how aging might differently affect frequency discrimination or detection of a change in frequency within a stimulus. In the present study, pure-tone frequency thresholds were measured in 19 older (61–74 years) and 20 younger (22–28 years) typically hearing adults. Participants were asked to discriminate between reference and probe frequencies or to detect changes in frequency within a probe stimulus. Broadband spectro-temporal pattern perception was also measured using the spectro-temporal modulated ripple test (SMRT). Frequency thresholds were significantly poorer in the basal than in the apical region of the cochlea; the deficit in the basal region was 2 times larger for the older than for the younger group. Frequency thresholds were significantly poorer in the older group, especially in the basal region where frequency detection thresholds were 3.9 times poorer for the older than for the younger group. SMRT thresholds were 1.5 times better for the younger than for the older group. Significant age effects were observed for SMRT thresholds and for frequency thresholds only in the basal region. SMRT thresholds were significantly correlated with frequency thresholds only in the older group. The poorer frequency and spectro-temporal pattern perception may contribute to age-related deficits in speech perception, even when audiometric thresholds are nearly normal.

## 1 Introduction

Compared to younger people, older people tend to have greater difficulty understanding speech in steady noise or in competing speech [[1], [2], [3], [4], [5], [6], [7], [8]], even when audiometric thresholds are normal or nearly normal. Auditory spectral resolution represents the ability to distinguish sounds in the frequency domain [9]. Poor spectral resolution can limit speech performance in adults with hearing loss, adults with cochlear implants, and adults with normal hearing listening to cochlear implant simulations [[10], [11], [12], [13], [14], [15], [16]].

Spectral resolution is ultimately limited by frequency perception. Frequency discrimination and discrimination thresholds are typically measured using pure tones. Frequency discrimination thresholds have been shown to decline with age [12,[17], [18], [19]], possibly due to broadening of auditory filters associated with aging [20,21]. Various psychophysical methods can be used to measure frequency thresholds. For example, frequency discrimination thresholds (FDTs) have been frequently used to characterize frequency perception [[22], [23], [24]]. In a FDT task, listeners must discriminate between differences in frequency in probe and reference stimuli. Typically, the probe and reference frequencies are applied to the entire duration of the stimuli. FDTs have been significantly associated with speech perception in normal-hearing and cochlear implant listeners [19]. Frequency perception can also be characterized in terms of detection of an abrupt change in frequency within a stimulus (“frequency change detection thresholds”, or FCDTs). There is evidence that different neural mechanisms are involved in perception of a frequency difference introduced at onset of a probe (as in FDTs) and a change in frequency embedded within the probe [25,26]. FCDTs have been measured in both behavioral and electrophysiological studies involving normal-hearing and cochlear implant listeners [[25], [26], [27], [28]]. FCDTs may better relate to listeners’ ability to perceive changes in frequency that occur in speech (e.g., vowel formant transitions) by minimizing the interference of probe onset cues [28]. Frequency glide discrimination thresholds (FGDTs) require detection of a gradual change in frequency embedded within the probe stimulus [29,30]. While FGDTs would be expected to relate to frequency transitions in speech (e.g., formant transitions), the relationship is somewhat unclear [31]. While some have argued that sensitivity to abrupt or gradual changes in frequency may be driven by different perceptual mechanisms [32], others have found that the same mechanism may underlie sensitivity to frequency changes, whether abrupt or smooth [33].

The above frequency threshold tasks are local measures of frequency perception. Broadband and/or band-limited spectral resolution, which presumably involves integration of frequency information across the cochlea, has often been characterized using spectral ripple detection and/or discrimination [14,[34], [35], [36], [37]]. Discriminating between different spectrally rippled broadband stimuli may be particularly relevant to speech recognition tasks because the ripples are distributed across a broad cochlear region. Also, perception of spectral peaks in rippled stimuli may relate to perception of spectral features in speech (e.g., vowel formant frequencies). Strong correlations have been observed between spectral ripple perception and phoneme recognition when data were pooled across normal-hearing, hard-of-hearing, and cochlear implant listeners [14]. The spectral ripple stimuli used in some previous studies [34,38] may have included loudness cues at the spectral edges of the stimuli that may have contributed to spectral ripple perception [39]. Listeners might also attend to a particular spectral region or to the spectral centroid in a spectral ripple perception task [39]. The spectral-temporal modulated ripple test (SMRT) [40,41] modified previous spectral ripple tests by incorporating a modulation phase that drifts over time, which theoretically would reduce local loudness cues and spectral edge-listening. As such, the SMRT might better reflect spectral resolution than previous spectral ripple tasks. The SMRT has been correlated with speech performance in normal-hearing, hard of hearing, and cochlear implant listeners [[42], [43], [44]]. However, SMRT thresholds may still be susceptible to perception of unintentional temporal cues [45,46]. We acknowledge the shortcomings and potential confounds in the SMRT and other spectral ripple perception tasks that may limit their ability to characterize broadband spectral resolution.

While age effects have been reported for frequency detection and discrimination, relatively few studies have examined age effects for broadband measures of spectral ripple perception. Sheft et al. [47] found that spectral ripple phase perception and speech understanding in noise were poorer in older than in younger adults. Nambi et al. [36] found that spectral ripple detection thresholds and speech understanding in noise were poorer in older than in younger adults. However, Ozmeral et al. [37] found no significant difference between older and younger listeners in terms of the “spectral modulation transfer function”, defined as the spectral modulation depth threshold as a function of modulation frequency.

The aim of the present study was to compare frequency discrimination/detection thresholds and broadband SMRT thresholds between younger and older typically hearing adults. While age-related deficits have been observed frequency discrimination [12,[17], [18], [19],^37^], it is unclear whether such deficits would persist for discrimination of changes in frequency within a stimulus. It is also unclear how age might affect broadband SMRT thresholds. In this study, we measured FDTs (frequency differences across reference and probe stimuli), FCDTs and FDGTs (frequency changes embedded within a probe stimulus), as well as SMRT thresholds in younger and older typically hearing adults. Note that in the FDT task, listeners must hold all three stimuli in working memory to detect the different frequency in the probe, while in the FCDT and FGDT tasks, listeners must simply detect a change in frequency within one of the intervals. As such, the FDT task requires working memory resources than do the FCDT and FGDT tasks. Accordingly, we hypothesized that aging would have a greater effect on FDTs than FCDTs or FGDTs, given the greater demands on working memory, which would be negatively affected by aging [48,49]. We also hypothesized that aging would affect broadband SMRT thresholds due to declines in central auditory processes [[50], [51], [52]].

## 2 Methods

### 2.1 Participants

Twenty young adults (10 females, 10 males) and 19 older adults (12 females, 7 males) participated in the study. All were native Chinese speakers of Mandarin. All participants were recruited from the First Affiliated Hospital of Soochow University in Suzhou, China. All procedures were conducted in accordance with the review and approval of the ethical committee at Soochow University, Suzhou, China (approval number: 2021041). Participants provided verbal and written consent prior to beginning the study and were compensated at a rate of 100 yuan per hour plus travel expenses, in accordance with departmental policy.

None of the participants reported any hearing problems, neurological disorders, or use of psychoactive medications. The Mini-Mental State Examination [53] was administered to the older participants, and indicated no abnormalities in cognitive function (all scores ≥28/30).

**Fig. 1:**
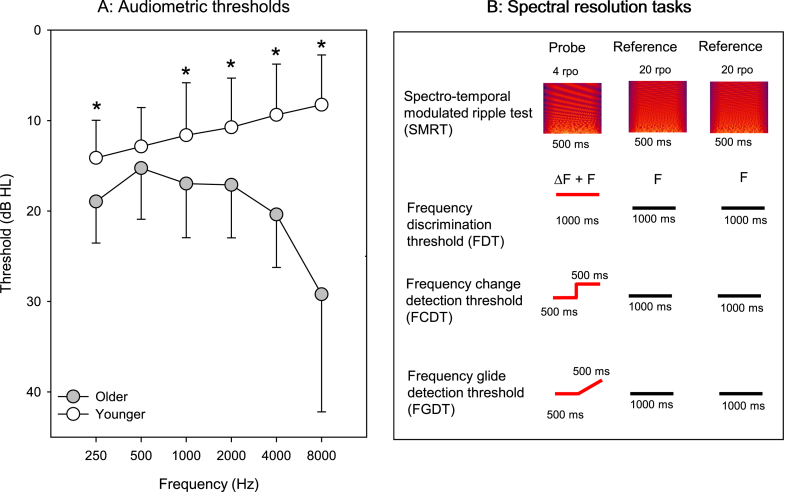
A: Mean audiometric thresholds and standard deviation for the older and younger participants. The error bars show the standard deviation, and the asterisks indicate significantly different audiometric thresholds between the younger and older groups (p < 0.05). B: Illustration of the stimuli for the spectral resolution tasks, the y-axis for all plots is frequency in Hertz. At top are shown the probe (4 ripples per octave, or rpo) and reference stimuli (20 rpo) for the SMRT. The duration of the SMRT stimuli was 500 ms. Below are the probe (red) and reference stimuli (black) for the frequency discrimination threshold (FDT), frequency change discrimination threshold (FCDT), and frequency glide discrimination threshold (FGDT) tasks. The duration for the frequency threshold stimuli was 1000 ms. For the FCDT and FGDT probe stimuli, the frequency was the same as the reference for the first 500 ms, then changed for the final 500 ms.

The mean age at testing ±standard deviation was 24.9 ± 2.3 and 67.2 ± 3.5 years for the younger and older participants, respectively. Because Wilcoxon signed rank tests showed no significant difference in pure-tone thresholds between the left and right ears at any audiometric frequency (p > 0.05 for all comparisons), threshold data were averaged across ears at each frequency. Fig. 1A shows the mean and standard deviation for audiometric thresholds for the older and younger participants. The audiograms for the older and younger groups are “age-typical,” since most thresholds did not exceed the range expected for 95% of the population based on age and gender (ISO 7029: 2017). A Student's t-test showed that pure-tone thresholds averaged across all audiometric frequencies (PTA thresholds) were significantly higher for the older than for younger group [t(37) = 6.4, p < 0.001]. T-tests also showed that thresholds were significantly higher for the older than for the younger group at 250 Hz [t(37) = 3.4, p = 0.001], 1000 Hz [t(37) = 2.8, p = 0.007], 2000 Hz [t(37) = 3.5, p = 0.001], 4000 Hz [t(37) = 6.0, p < 0.001], and 8000 Hz [t(37) = 6.6, p < 0.001], but not at 500 Hz [t(37) = 1.5, p = 0.145].

### 2.2 Methods

None of the participants had any previous experience with the experimental tests. Prior to formal testing, multiple practice trials were conducted to familiarize participants with the stimuli and procedures. All stimuli were presented via headphones (Sennheiser I 280) at 65 dBA. The experimental stimuli are illustrated in Fig. 1B. Thresholds for the FCD, FCDT, FGDT, and SMRT tasks were measured in separate test blocks, and the test blocks were randomized and counter-balanced within and across participants.

#### 2.2.1 Fixed frequency discrimination thresholds (FDTs)

FDTs were measured using Mandarin Angel Sound Training software (http://mast.emilyfufoundation.org). A 3-alternative forced-choice (3AFC) adaptive paradigm was used (2-down/1-up), converging on the frequency difference that produced 71% correct on the psychometric function [54]. Each test trial consisted of three intervals; the inter-stimulus interval was 500 ms. Two of the intervals contained the reference stimuli and the third contained the probe (Fig. 1B); the interval order was randomized across trials. The duration of the reference and probe stimuli was 1000 ms. The reference stimuli were 500-Hz or 4000-Hz pure tones. The 500- and 4000-Hz reference frequencies were chosen to target relatively apical and basal regions of the cochlea. The 500-Hz reference was partly within the limits of temporal pitch perception [[55], [56], [57]], while the 4000-Hz reference frequency was above the limit of temporal pitch perception. Finally, the 500-Hz reference was within the range of vowel formant frequencies, while the 4000-Hz reference was above this range. As such, the 500- and 4000-Hz reference frequencies represented different cochlear regions, different contributions of temporal cues, and different contributions to speech perception. The probe stimuli were pure tones that were ≥ the reference frequency. A 20-ms amplitude ramp and damp was applied to each stimulus to prevent click-like sounds at the beginning and end of the stimulus.

During testing, the three intervals were presented, and the participant responded by clicking on the interval that was different. The initial frequency of the probe was 16% higher than the reference frequency. The probe frequency was adjusted according to the correctness of the response. If the listener responded correctly two times in a row, the probe frequency was reduced; if the listener responded incorrectly 1 time, the probe frequency was increased. During each test run, the step size was adjusted according to the frequency difference between the reference and probe stimuli. When the frequency difference between the reference and probe stimuli was between 0.0% and 0.5%, the step size was 0.1% of the reference frequency. Thus, the step size in this range was 0.5 Hz for the 500-Hz reference and 4 Hz for the 4000-Hz reference. When the frequency difference between the reference and probe stimuli was between 0.5% and 2.0%, the step size was 0.5% of the reference frequency. Thus, the step size in this range was 2.5 Hz for the 500-Hz reference and 20 Hz for the 4000-Hz reference. When the frequency difference between the reference and probe stimuli was >2.0%, the step size was 2.0% of the reference frequency. Thus, the step size in this range was 10 Hz for the 500-Hz reference and 80 Hz for the 4000-Hz reference. The adaptive run was terminated after 35 trials, with a minimum of 6 reversals in probe frequency; if there were less than 6 reversals, the test run was discarded and then repeated. The discrimination threshold at each reference frequency was calculated as the average of the last 6 reversals. Two test runs were conducted for each of the frequency discrimination measures, and thresholds were averaged across the two runs.

#### 2.2.2 Dynamic frequency change detection thresholds (FCDTs)

FCDTs were measured using Mandarin Angel Sound Training software (http://mast.emilyfufoundation.org). A 3AFC adaptive paradigm was used (2-down/1-up); note that only a descending staircase was used. Each test trial consisted of three intervals; the inter-stimulus interval was 500 ms. The duration of the reference and probe stimuli was 1000 ms. The reference stimuli were 500-Hz or 4000-Hz pure tones. For the probe stimuli, the frequency for the first 500 ms was the same as the reference frequency, followed by an abrupt change in frequency that was ≥ the reference frequency for the final 500 ms (Fig. 1B). The transition at 500 ms occurred at 0° phase (zero crossing) to prevent audible transient clicks [25]. A 20-ms amplitude ramp and damp was applied to each stimulus to prevent click-like sounds at the beginning and end of the stimulus.

During testing, the three intervals were presented, and the participant responded by clicking on the interval that was different. The initial frequency of the probe was 16% higher than the reference frequency. The probe frequency was adjusted according to the correctness of the response. If the listener correctly responded two times in a row, the probe frequency was reduced; if the listener responded incorrectly 1 time, the probe frequency was increased. During each test run, the step size was adjusted according to the frequency difference between the reference and probe stimuli. When the frequency difference between the reference and probe stimuli was between 0.0% and 0.5%, the step size was 0.1% of the reference frequency. Thus, the step size in this range was 0.5 Hz for the 500-Hz reference and 4 Hz for the 4000-Hz reference. When the frequency difference between the reference and probe stimuli was between 0.5% and 2.0%, the step size was 0.5% of the reference frequency. Thus, the step size in this range was 2.5 Hz for the 500-Hz reference and 20 Hz for the 4000-Hz reference. When the frequency difference between the reference and probe stimuli was >2.0%, the step size was 2.0% of the reference frequency. Thus, the step size in this range was 10 Hz for the 500-Hz reference and 80 Hz for the 4000-Hz reference. The adaptive run was terminated after 35 trials, with a minimum of 6 reversals in probe frequency; if there were less than 6 reversals, the test run was discarded and then repeated. The discrimination threshold at each reference frequency was calculated as the average of the last 6 reversals. Two test runs were conducted for each of the frequency discrimination measures, and thresholds were averaged across the two runs.

#### 2.2.3 Dynamic frequency glide detection thresholds (FGDTs)

FGDTs were measured using Mandarin Angel Sound Training software (http://mast.emilyfufoundation.org). A 3AFC adaptive paradigm was used (2-down/1-up); note that only a descending staircase was used. Each test trial consisted of three intervals; the inter-stimulus interval was 500 ms. The duration of the reference and probe stimuli was 1000 ms. The reference stimuli were 500-Hz or 4000-Hz pure tones. For the probe stimuli, the frequency for the first 500 ms was the same as the reference frequency, followed by a gradual change in frequency over the final 500 ms, where the final frequency was ≥ the reference frequency (Fig. 1B). The transition at 500 ms occurred at 0° phase (zero crossing) to prevent audible transient clicks [25]. A 20-ms amplitude ramp and damp was applied to each stimulus to prevent click-like sounds at the beginning and end of the stimulus.

During testing, the three intervals were presented, and the participant responded by clicking on the interval that was different. The initial frequency of the probe was 16% higher than the reference frequency. The probe frequency was adjusted according to the correctness of the response. If the listener correctly responded two times in a row, the probe frequency was reduced; if the listener responded incorrectly 1 time, the probe frequency was increased. During each test run, the step size was adjusted according to the frequency difference between the reference and probe stimuli. When the frequency difference between the reference and probe stimuli was between 0.0% and 0.5%, the step size was 0.1% of the reference frequency. Thus, the step size in this range was 0.5 Hz for the 500-Hz reference and 4 Hz for the 4000-Hz reference. When the frequency difference between the reference and probe stimuli was between 0.5% and 2.0%, the step size was 0.5% of the reference frequency. Thus, the step size in this range was 2.5 Hz for the 500-Hz reference and 20 Hz for the 4000-Hz reference. When the frequency difference between the reference and probe stimuli was >2.0%, the step size was 2.0% of the reference frequency. Thus, the step size in this range was 10 Hz for the 500-Hz reference and 80 Hz for the 4000-Hz reference. The adaptive run was terminated after 35 trials, with a minimum of 6 reversals in probe frequency; if there were less than 6 reversals, the test run was discarded and then repeated. The discrimination threshold at each reference frequency was calculated as the average of the last 6 reversals. Two test runs were conducted for each of the frequency discrimination measures, and thresholds were averaged across the two runs.

#### 2.2.4 Spectro-temporally modulated ripple test (SMRT)

SMRT thresholds were measured using Mandarin Angel Sound Training software (http://mast.emilyfufoundation.org). The SMRT stimuli were 500 ms in duration with 100-ms onset and offset linear ramps. A 3AFC paradigm (1-up/1-down) adaptive procedure was used to measure ripple frequency threshold discrimination threshold in terms of ripples per octave (rpo), converging on a 50% discrimination threshold. As such, all reference and probe stimuli were spectro-temporally modulated. Note that this is different from a ripple detection task, where a fixed rpo is used for the probe and the reference stimuli are unmodulated, and the listener must detect the presence of a spectral ripple. For the present study, during each trial, three rippled noises were presented across intervals. The inter-stimulus interval was 500 ms. Two intervals contained the reference stimuli, with 20 rpo density; the third interval contained the probe stimulus, with a rpo density that was ≤ the reference rpo density. Listeners were asked to identify the probe, and the probe ripple density was adapted according to the correctness of the response. The initial density of the probe stimulus was 0.5 rpo and the step size was 0.2 rpo. The order of the reference and probe stimuli was randomized across trials. The adaptive run was terminated after eight reversals; the last 6 reversals were used to calculate the mean. Two test runs were conducted, and SMRT thresholds averaged across the two runs. SMRT thresholds were expressed in terms of rpo.

## 3 Results

**Fig. 2:**
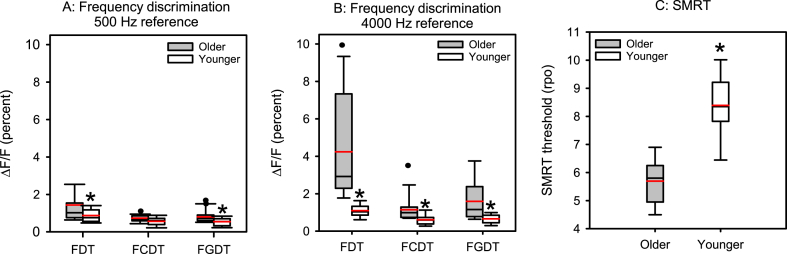
A. Boxplot of frequency thresholds in percent for the frequency discrimination threshold (FDT), frequency change discrimination threshold (FCDT), and the frequency glide discrimination threshold (FGDT) with the 500 Hz reference, for the older and younger groups. B. Same as A, but for the 4000 Hz reference. C. Boxplot of SMRT thresholds in ripples per octave (rpo) for the older and younger groups. In all panels, the boxes show the 25th and 75th percentiles, the error bars show the 10th and 90th percentiles, the black horizontal line shows the median, the red horizontal line shows the mean, the black circles show outliers, and the asterisks indicate significantly different thresholds between the younger and older groups (p < 0.05).

Fig. 2A and B shows boxplots of FDTs, FCDTs, and FGDTs in percent for the 500 Hz and 4000 Hz reference frequencies, for older and younger participants; mean data are shown in Table 1. In general, discrimination thresholds were lower (better) for the younger than the older group, lower with the 500 Hz than the 4000 Hz reference, and lower for dynamic than for fixed changes in frequency. Because the distributions of the frequency threshold data violated assumptions of normality, non-parametric tests were used for analysis. Across all tests and all participants, a Mann-Whitney rank sum test showed that frequency detection and discrimination thresholds were significantly higher at 500 Hz than at 4000 Hz [U = 4417, p < 0.001). Across both reference frequencies and all participants, a Kruskal-Wallis analysis of variance on ranked data showed a significant effect of test (FDT, FCDT, FGDT) on frequency thresholds [H(2) = 45.5, p < 0.001); post-hoc Tukey tests showed that frequency thresholds were significantly higher for the FDT than for the FCDT and FGDT tests (p < 0.001 for both comparisons), with no significant difference between the FCDT and FGDT tests. Mann-Whitney rank sum tests showed that thresholds were significantly higher for the older than for the younger group at 500 Hz for FDTs (p = 0.003) and FGDTs (p = 0.022), and at 4000 Hz for FDTs (p < 0.001), FCDTs (p < 0.001), and FGDTs (p < 0.001). Fig. 2C shows box plots of SMRT thresholds in rpo; mean thresholds are shown in Table 1. A Student's t-test showed that SMRT thresholds were significantly lower (poorer) for the older than for the younger group [t(37) = −9.3, p < 0.001).

**Table 1:** Mean and standard deviation for frequency thresholds (ΔF/F in %) at 500 Hz and 4000 Hz and spectro-temporal modulated ripple test (SMRT) thresholds for the older and younger groups. Frequency thresholds are shown for the frequency discrimination threshold (FDT), frequency change discrimination thresholds (FCDT), and the frequency glide discrimination threshold (FGDT) tasks. Results for Mann-Whitney rank sum tests comparing the older and younger groups is shown at right.

		Older	Younger	Statistics
Frequency threshold at 500 Hz (ΔF/F in %)	FDT	1.44 ± 1.28	0.87 ± 0.35	U = 455.5, T = 655.5, p = 0.003*
	FCDT	0.73 ± 0.26	0.57 ± 0.21	U = 123.5, T = 446.5, p = 0.064
	FGDT	0.80 ± 0.36	0.54 ± 0.23	U = 108.0, T = 462.0, p = 0.002*
Frequency threshold at 4000 Hz (ΔF/F in %)	FDT	4.24 ± 2.87	1.10 ± 0.35	U = 5.5, T = 564.5, p < 0.001*
	FCDT	1.15 ± 0.62	0.61 ± 0.28	U = 57.5, T = 512.5, p < 0.001*
	FGDT	1.59 ± 1.05	0.66 ± 0.28	U = 57.5, T = 512.5, p < 0.001*
SMRT threshold (rpo)		5.69 ± 0.87	8.53 ± 1.02	U = 5.5, T = 195.5, p < 0.001*

To control for potential audibility differences between the older and younger groups, age effects were also calculated using Quade's non-parametric analysis of covariance; the non-parametric test was used because of violations of normal distributions. First, data were ranked for FDTs, FCDTs, and FGDTs at 500 and 4000 Hz, SMRT thresholds, and PTA thresholds across 500, 1000, 2000, and 4000 Hz. Next, linear regressions were performed between the ranked PTA data and the ranked frequency discrimination and SMRT data. The residuals from these regressions were used as the dependent variables for analyses of variance, with group (younger, older) as the fixed factor. Results showed no significant age effects for FDTs, FCDTs, or FGDTs at 500 Hz, but significant age effects for FDTs at 4000 Hz [F(1,37) = 23.9, p < 0.001], FCDTs at 4000 Hz [F(1,37) = 5.6, p = 0.024], [F(1,37) = 7.9, p = 0.008], and SMRT thresholds [F(1,37) = 23.9, p < 0.001].

Frequency thresholds (log scale) were also compared to SMRT thresholds; results of Pearson correlation analyses are shown in Table 2. Across all participants, significant associations were observed between SMRT thresholds and FDTs, FCDTs, and FGDTs at 500 Hz and 4000 Hz, even after Bonferroni adjustment for multiple comparisons (p < 0.008 for all comparisons). For the older group, significant associations were observed between SMRT thresholds and FDTs and FGDTs at 500 Hz, as well as FDTs, FCDTs, and FGDTs 4000 Hz (p < 0.05). After Bonferroni adjustment for multiple comparisons (p < 0.008), a significant association persisted only between SMRT thresholds and FCDTs at 4000 Hz. No significant associations were observed for the younger group. Because there was great inter-correlation among the FDTs, FCDTs, and FGDTs within each base frequency, the frequency threshold data were reduced using principal component analysis (PCA). Within each of the reference conditions, data were collapsed into to 1 factor, which explained 78.8% and 86.0% of the variance in the 500-Hz and 4000-Hz reference conditions, respectively. The values extracted from the PCA were then compared to SMRT thresholds using Pearson correlation analyses. Across all participants, significant correlations were observed between SMRT thresholds and the 500-Hz (r = −0.57, p < 0.001) and 4000-Hz data (r = −0.76, p < 0.001). Within the older group, significant correlations were observed between SMRT thresholds and the 500-Hz (r = −0.52, p = 0.022) and 4000-Hz data (r = −0.65, p = 0.003). No significant associations were observed for the younger group.

**Table 2:** Correlation coefficients for Pearson correlations between SMRT thresholds, FDTs, FCDTs, and FGDTs at 500 Hz and 4000 Hz, for older, younger, and all participants. Italics indicate significant associations; * = p < 0.05; ** = p < 0.008 (Bonferroni adjustment for multiple comparisons).

	500 Hz			4000 Hz		
Group	FDT	FCDT	FGDT	FDT	FCDT	FGDT
Older (n = 19)	−0.46*	−0.39	−0.52*	−0.55*	−0.64**	−0.56*
Younger (n = 20)	−0.43	−0.27	−0.35	−0.17	−0.17	−0.24
All n = 39)	−0.53**	−0.46**	−0.54**	−0.79**	−0.65**	−0.68**

## 4 Discussion

Frequency detection and discrimination thresholds (in percent) were poorer in the basal (4000-Hz reference) than in the apical region of the cochlea (500 Hz). These data are not consistent with previous studies that show poorer frequency detection and discrimination thresholds for relatively low than high base frequencies [19,58,59]. It is possible that the lifelong experience with a tonal language (Mandarin Chinese) in the present participants may have benefited frequency perception in the apical region [[60], [61], [62]].

Significant age effects were observed for frequency thresholds at 500 Hz and 4000 Hz. Note that audiometric thresholds were significantly higher for the older than for the younger group for all audiometric frequencies except for 500 Hz (see Fig. 1A). Divenyi and Haupt [11] suggested that even mild hearing loss may negatively affect psychoacoustic performance. After controlling for PTA thresholds across 500, 1000, 2000, and 4000 Hz, significant age effects persisted for frequency thresholds at 4000 Hz.

Across all tests, mean frequency thresholds were 2.4 times poorer for the older than for the younger group at 4000 Hz, and 1.2 times poorer at 500 Hz. The greater age effects at 4000 Hz are not consistent with previous studies that showed greater age effects at relatively low frequencies [19,58,59], presumably due to age-related declines in phase locking. Again, tonal language experience may have benefitted frequency perception at 500 Hz. The results are somewhat consistent with Clinard et al. [63], who found that both FDTs and frequency following responses (FFRs) declined with age, with poorer discrimination performance at 1000 Hz than at 500 Hz. Clinard and Cotter [64] reported that FFRs for frequency sweeps were more degraded in older than in younger adults, consistent with the present FCDT and FGDT stimuli.

Frequency thresholds were significantly poorer for fixed (FDT) than for dynamic changes in frequency (FCDTs, FGDTs). The present data are consistent with previous studies that reported poorer perception of fixed than dynamic changes in frequency [25,26]. There was no significant difference between FCDTs and FGDTs, consistent with Demany et al. [33] who suggested that similar mechanisms may underlie perception of a gradual of abrupt change in frequency. However, the data are not consistent with Tyler et al. [65], who found poorer discrimination thresholds for dynamic than for fixed changes in frequency. Note that the 50-ms stimulus duration used in Tyler et al. [65] was much shorter than the 1000-ms duration used in the present study, which may have affected outcomes.

Mean fixed thresholds (FDTs) were 3.9 times poorer for the older than for the younger group at 4000 Hz, and 1.7 times poorer at 500 Hz. With dynamic changes in frequency (averaged across FCDTs and FGDTs), mean thresholds were 2.2 times poorer for the older than for the younger group at 4000 Hz, and 1.4 times poorer at 500 Hz. The discrepancy in thresholds between fixed and dynamic changes in frequency was much larger for older listeners with the 4000 Hz reference. Again, greater working memory demands for the FDTs and tonal language benefits at 500 Hz may have partly contributed to the greater age-related deficits for perception of fixed changes in frequency in the basal region.

The frequency detection and discrimination tasks likely differed in terms of cognitive load. The FDT task required that all three intervals be held in short-term memory, while the FCDT and FGDT tasks required only discrimination of a change in frequency in one of the three intervals. Zhang et al. [66] found a significant correlation between backward digit span (a measure of working memory) and FDTs when the reference frequency was roved from trial to trial. However, Mishra and Dey [67] found no significant relationship between FDTs and forward or backward digit span in adults with normal hearing or with unilateral hearing loss.

Across all participants, mean SMRT thresholds were 1.5 times higher (better) for the younger than for the older group. The present SMRT results are consistent with Nambi et al. [36], who found significant age effects for spectral ripple thresholds, but not consistent with Ozmeral et al. [37], who found no significant age effects in terms of the spectral modulation transfer function. Note that the present SMRT paradigm required discrimination of spectro-temporally modulated stimuli that differed in terms of rpo, whereas Ozmeral et al. [37] measured detection of spectral modulation at different fixed rpos. Note that Ozmeral et al. [37] used spectrally rippled noise, rather than the sine-wave SMRT stimuli used in the present study. In the present study, the age-related deficit was much larger for FDTs at 4000 Hz than for SMRT thresholds, suggesting that frequency discrimination in the basal region may be more sensitive to age effects than SMRT thresholds.

Across all participants SMRT thresholds were significantly correlated with frequency thresholds (Table 2), due to age effects. SMRT thresholds were significantly correlated with frequency thresholds in the older group, but not in the younger group. The strongest correlation within the older group was observed between SMRT thresholds and FCDTs at 4000 Hz. When frequency threshold data were combined within the 500 Hz and 4000 Hz base frequency conditions (as a more general measure of frequency perception), significant correlations were observed between SMRT thresholds and frequency threshold data only for the older group. The correlation was stronger for the 4000-Hz than for the 500-Hz frequency data, consistent with the much larger deficit in frequency thresholds observed at 4000 Hz than at 500 Hz reference (Fig. 2). However, the frequency threshold data only partly explained the variability in SMRT thresholds (r^2^ = 0.27 for the 500-Hz data; r^2^ = 0.42 for the 4000 Hz data). This suggests that sensitivity to some other components in the SMRT stimuli may have contributed to SMRT thresholds. Previous studies have suggested that unintentional temporal cues may contribute to SMRT thresholds [45,46]. Narne et al. [45] found that the outputs of the auditory filters above 6400 Hz (the upper frequency edge of the spectral ripple stimuli) may have introduced unintentional temporal cues that may have contributed to spectral ripple frequency discrimination thresholds; using notched noise at the edges of the spectral ripple stimuli eliminated these cues. In the present study, differences in high frequency thresholds between the older and younger groups (Fig. 1A) may have resulted in different access to these unintentional temporal cues and may partly explain difference in SMRT thresholds between the groups. It is also unclear whether the SMRT truly reflects integration of frequency information across the selected bandwidth or is driven by local frequency sensitivity. For example, Narne et al. [68] found no significant difference in spectral ripple glide thresholds between narrowband and broadband ripple stimuli, suggesting that frequency information may not be combined across the cochlea. In the present study, only broadband SMRT thresholds were measured.

To the extent that SMRT thresholds reflect spectral resolution, it is tempting to interpret associations between SMRT and frequency thresholds as indicative of integration of frequency information across the cochlea, at least in the older group. Peters and Moore [69] found that when audiometric thresholds in older listeners were within 20 dB of normal, their auditory filters were also in the normal range. Moore and Peters [19] showed that frequency detection and discrimination thresholds were unrelated to auditory filter bandwidth [19]. Taken together, these studies suggest that frequency detection and discrimination thresholds may be unrelated to frequency selectivity. Unfortunately, frequency selectivity was not measured in the present study, so it is difficult to assess how frequency discrimination and detection, spectral resolution, and SMRT thresholds may inter-relate, especially in older listeners.

## 5 Conclusions

Frequency detection and discrimination thresholds at 500 Hz or 4000 Hz and SMRT thresholds were measured in older and younger typically hearing adults. After controlling for audibility, there was no significant difference between groups for frequency thresholds at 500 Hz; however, frequency thresholds at 4000 Hz and SMRT thresholds were significantly poorer in older than in younger listeners. Across all listeners and both reference frequencies, discrimination thresholds were significantly lower for dynamic than for fixed changes in frequency. A significant relationship was observed between SMRT thresholds and frequency thresholds in the older group, but not in the younger group. The age-related deficit was much larger for FDTs at 4000 Hz than for SMRT thresholds, suggesting that FDTs in the basal region may be more sensitive to age effects than broadband SMRT thresholds. The age-related deficits in frequency detection, frequency discrimination and spectro-temporal pattern perception may contribute to the difficulties in masked speech perception observed in older adults, despite having nearly normal hearing.

## Funding

This work was supported by the 10.13039/501100001809National Natural Science Foundation of China (81970877&82171159), the 10.13039/501100004608Jiangsu Provincial Natural Science Foundation - Outstanding Youth Foundation (BK20200054).

## Ethics statement

The studies involving human participants were reviewed and approved by the Ethical Committee from the First Affiliated Hospital of Soochow University (Approval number 2021041). All participants provided written informed consent before participating in the study.

## Author contribution statement

Duo-Duo Tao: Conceived and designed the experiments; Analyzed and interpreted the data; Contributed reagents, materials, analysis tools or data; Wrote the paper.

Bin Shi: Performed the experiments; Analyzed and interpreted the data; Wrote the paper.

John J. Galvin: Analyzed and interpreted the data; Contributed reagents, materials, analysis tools or data; Wrote the paper.

Ji-Sheng Liu; Qian-Jie Fu: Conceived and designed the experiments; Contributed reagents, materials, analysis tools or data; Wrote the paper.

## Data availability statement

Data included in article/supplementary material/referenced in article.

## Additional information

Supplementary content related to this article has been published online at [URL].

## Declaration of competing interest

The authors declare that they have no known competing financial interests or personal relationships that could have appeared to influence the work reported in this paper.
